# Barriers in Opting for Emergency Medicine as a Career in Pakistan: A Cross-Sectional Survey Study

**DOI:** 10.7759/cureus.52525

**Published:** 2024-01-18

**Authors:** Abdul Mutaal, Saad Bajwa, Muhammad Saad ur Rehman, Khubaib Ashiq, Fahad Shuaib, Dawood Shehzad, Mohammed Ali Sufiyan, Bakhtawar Yasmeen, Waseem Abbas, Zaeema Noor

**Affiliations:** 1 Plastic and Reconstructive Surgery, Rawalpindi Medical University, Rawalpindi, PAK; 2 Internal Medicine, Rawalpindi Medical University, Rawalpindi, PAK; 3 Ophthalmology, Mayo Hospital, Lahore, PAK; 4 Emergency Medicine, Rawalpindi Medical University, Rawalpindi, PAK; 5 Internal Medicine, University of South Dakota, Vermillion, USA; 6 Family Medicine, Rawalpindi Medical University, Rawalpindi, PAK; 7 Medicine, Rawalpindi Medical University, Rawalpindi, PAK

**Keywords:** emergency medicine barriers, career barriers, perceived barriers, emergency medicine, career option

## Abstract

Background: Medical professionals' low level of apprehension and insights may result in the undervaluing of emergency medicine (EM) as a speciality to pursue in the future, which is a vital component in the everyday management of hundreds of patients.

Aim: The aim of this study is to assess medical professionals' perception of the barriers in opting for EM as a career option in Pakistan.

Method: This was an online survey study that examined doctors'/medical students’ perception of hurdles in EM in Pakistan between November and December 2023. This study was conducted at Rawalpindi Medical University, Rawalpindi, Pakistan. Social media platforms were used to recruit the participants to carry out this survey. The questionnaire proforma comprised three sections: a demographic characteristics section (six questions), a perception section (11 questions), and the last section, where participants were asked to give their opinion to improve EM for a future speciality.

Results: An online Google survey form was used for the acquisition of data. Percentage and frequency distribution analysis was used. A total of 144 individuals (N = 144) participated in this study. Around 33.3% (N = 48) of the participants expressed that they had not considered a career in EM. Around 43% (N = 62) of them reported not having done a clinical placement in this speciality. A majority of the participants said that either they or their close friends/family members had faced a medical emergency. Leading barriers that proved a hindrance in pursuing this field were high levels of burnout, poor work-life balance, loss of patient follow-up, inability to work independently, more hostile environment, increased mortalities, and lack of exposure. Interestingly, family pressure had been reported by some participants as a limiting factor in pursuing EM.

Conclusion: In Pakistan, many doctors and medical students are not willing to pursue their careers in EM. Nationwide educational seminars should be conducted to increase awareness and interest among doctors in this field. Moreover, more and more clinical placement opportunities should be made available for junior doctors in EM. In the future, further research should be carried out to identify effective educational interventions to increase doctors’/medical students’ awareness in this field of medicine.

## Introduction

An emergency department provides remedies to patients with complex conditions and diagnoses in a wide range of diseases and comorbidities [1]. Contrary to all the other fields, it has to provide care to patients that span the spectrum of all medical specialties [1]. The emergency health care system remains the core medical safety net for all the people needing care in any country [2]. The role of emergency physicians (EPs) was reported by the American College of Emergency Physicians in 1994 as the first exposure provider to people regardless of their age, gender, time of presentation, and ability to afford [2]. Several barriers exist in emergency medicine (EM), but they are not usually reported [3]. One of the barriers is the unreported prevalence of gender and sexual harassment [3].

Africa contributes a great proportion of the total world population and thus has a significant disease and trauma burden. Less than 1% of total research works in Africa have been carried out in emergency settings [4]. Many internal factors, such as restricted supervision, inadequate facilities, and physician burnout, are some of the reported hindrances faced by physicians in Africa [4]. Research in the EM space follows a similar narrative in Pakistan.

Physician burnout is a grave hazard not only to the patient’s care but also to the physician’s quality of life [5]. This problem prevails in all the medical fields but is, in particular, more common in EM [5]. The effect of burnout causes EPs to have decreased clinical efficiency and more errors in medical judgment and even significantly increases their chances of suicide [6]. These effects are reported to be significantly higher in EM residents compared to medical residents of other specialties in the United States [6]. In Pakistan, there is a lack of awareness regarding pursuing EM as a field of specialization [7].

## Materials and methods

Study design

This study was conducted at Rawalpindi Medical University, Rawalpindi, Pakistan. An online cross-sectional survey using a self-developed questionnaire form was used to examine doctors’ and medical students’ perceptions of EM in Pakistan between November and December 2023 (see Appendix).

Study population

Our study population comprised doctors and final-year medical students in our tertiary care hospital who were over 18 years of age and were currently living in the country. There were no exclusion criteria. 

Data collection

A convenient sampling technique was used to collect the data. Social media platforms, including Facebook and WhatsApp, were used to invite the participants for the collection of required data. The cover page of the study indicated the objective of the study, and the participants were informed that proceeding with the proforma would be considered equivalent to informed consent.

The Ethical Review Board of Rawalpindi Medical University issued approval (reference number: 3742/MER/HFH). 

Study tool and pilot phase

The researcher developed a questionnaire tool to examine doctors’ and medical students’ perceptions of EM. The questionnaire tool was developed based on an extensive literature review. The total number of questions was 18. The questionnaire tool comprised three sections: a sociodemographic characteristic section (six questions), a perception section (11 questions), and a section where participants were asked to give their opinions (one question).

The face validity of the questionnaire was checked by expert clinicians from the Faculty of Emergency Medicine in Holy Family Hospital, Pakistan, and they confirmed the relevance and comprehensibility of the items in the questionnaire. To check the understandability of the questionnaire tool, we conducted a pilot study on a small group of members of doctors before distributing it on a larger scale. This was performed after expert clinicians checked the questionnaire tool.

Sample size calculation

The OpenEpi calculator (www.OpenEpi.com) was used to obtain the sample size. A confidence interval of 95%, a standard deviation (SD) of 0.5, and a margin of error of 5% were used, which gave the required sample size as 80 (N = 80).

## Results

Participants’ demographic information

A total of 144 individuals participated in this study. Around 50.7% (N = 73) of them were males and 58.3% (N = 84) were employed as a medical officer. Only 56.9% (N = 82) of them have undergone a clinical placement in EM. Around 70.1%(N = 101) of the participants had a close friend or a family member who had experience in EM. Meanwhile, 45.1% (N = 65) of the participants had faced a medical emergency by themselves. Interestingly, only 44.4% (N = 64) of the participants considered a career in EM. Table 1 presents the participants’ demographic characteristics.

**Table 1:** Participants’ demographic characteristics (N) indicates the number of participants (frequency) with a specific given response. (%) indicates the percentage of the participants with a specific given response.

Variable	Percentage (%)	Frequency (N)
Gender		
Male	50.7%	73
Female	48.6%	70
Prefer not to say	0.7%	01
Current designation		
Medical officer	58.3%	84
Medical student	9%	13
Postgraduate resident	9%	13
House officers	15.3%	22
Others	8.3%	12
Clinical placement in emergency medicine		
Yes	56.9%	82
No	43.1%	62
Any medical emergency in close friends or family members		
Yes	70.1%	101
No	25%	36
Prefer not to say	4.9%	07
Personal experience of any medical emergency		
Yes	45.1%	65
No	52.1%	75
Prefer not to say	2.8%	04
Career choice in emergency medicine		
Yes	44.4%	64
No	33.3%	48
Maybe	22.2%	32

Perception of barriers in EM

Table 2 presents the participants' responses to perception questions concerning EM as a potential future career. The biggest perceived barrier is a high level of burnout in EM at 86.1% of participants (N = 124), followed by poor work-life balance (72.2%, N = 104) and loss of patient follow-up (63.9%, N = 92). Eighty-six participants (59.7%) believed that emergency specialists could not work independently, while 83 (57.6%) had an opinion that EM offers a more hostile environment to work in. Seventy-seven (53.5%) of the participants recognized EM as less satisfying due to the increased number of mortalities and severely sick patients. Meanwhile, 41% (N = 59) acknowledged a lack of exposure to EM to consider it as a career. Surprisingly, 29.2% (N = 42) of the participants expressed that EM does not offer any further fellowship opportunities, in addition to the 27.8% (N = 40) of people who believe that emergency specialists earn less than other physicians. An unexpected response was from 15.3% (N = 22) of the participants who felt that their parents would not allow their career in EM, which also shows that family influence and peer pressure can be a deciding factor in opting for a career option in Pakistan.

**Table 2:** Participants' responses to perception questions concerning EM as a potential future career (N) indicates the number of participants (frequency) with a specific given response. (%) indicates the percentage of the participants with a specific given response.

Number	Variable	Percentage (%)	Frequency (N)
1.	I lack exposure to emergency medicine to make it my career choice.		
	Agree	41%	59
	Disagree	39.6%	57
	Neutral	19.4%	28
2.	I feel emergency specialists earn less than other fellows.		
	Agree	27.8%	40
	Disagree	46.5%	67
	Neutral	25.7%	37
3.	I feel doctors working in emergency medicine face high levels of burnout.		
	Agree	86.1%	124
	Disagree	6.3%	09
	Neutral	7.6%	11
4.	I feel emergency medicine offers a poor work-life balance.		
	Agree	72.2%	104
	Disagree	16.7%	24
	Neutral	11.1%	16
6.	I feel emergency medicine offers a more hostile environment.		
	Agree	57.6%	83
	Disagree	22.2%	32
	Neutral	20.1%	29
7.	I feel emergency medicine does not offer further specialization.		
	Agree	29.2%	42
	Disagree	43.1%	62
	Neutral	27.8%	40
8.	I feel emergency specialists cannot work independently.		
	Agree	59.7%	86
	Disagree	27.8%	40
	Neutral	12.5%	18
9.	I feel emergency medicine is less satisfying due to increased mortalities and severely sick patients.		
	Agree	53.5%	77
	Disagree	34.7%	50
	Neutral	11.8%	17
10.	I feel my family will not approve of my career in emergency medicine.		
	Agree	15.3%	22
	Disagree	40.3%	58
	Strongly disagree	22.9%	33
	Neutral	21.5%	31
11.	I feel emergency medicine is less rewarding in terms of patient follow-up.		
	Agree	63.9%	92
	Disagree	21.5%	31
	Neutral	14.6%	21

Participants’ opinions

The majority of the participants expressed their opinions on how can medical students and doctors be motivated to opt for EM as a potential career in the future. Some of the responses are given as follows:

"By proper exposure, workshops, and seminars. Also, by sharing reviews of people who are working/have specialization in emergency medicine." (Participant no. 1)

"Supervised learning, good security measures for a better working environment, regular therapy sessions, and enough breaks to lessen burnout." (Participant no. 2)

"To conduct workshops or seminars about emergency medicine where all future possibilities and outcomes should be discussed." (Participant no. 3)

"By providing career counseling regarding this specialty and a hands-on rotation in the department." (Participant no. 4)

"Emergency medicine physicians face higher burnout due to increased working hours and lower salaries than their colleagues working in other specialties. The healthcare system should start working for the benefit of doctors as well." (Participant no. 5)

"Higher salaries and fewer working hours create a work-life balance. I think this would develop the interest of junior doctors." (Participant no. 6)

## Discussion

The key findings of this study are as follows: 1) a considerable proportion of Pakistani doctors/medical students reported that they had not considered a career in EM, 2) a considerable proportion of the participants reported that they had not undergone any clinical internship in EM, and 3) several barriers exist that participants believe are a hindrance in pursuing a career in EM.

Our study suggests that a major factor that stops participants from opting for EM is a high level of burnout. Physicians and nurses giving EM services have high work demands, more responsibilities and uneasiness at work, and less margin for error [8]. Burnout has serious mental and physical effects on doctors and nurses working in an emergency; however, it is frequently dismissed [8]. An Irish study done in 2019 shows that Irish-trained doctors leave emergency training in their home country and join the Australian training program in non-EM-related fields due to burnout [9]. A significant portion (71%) of those reported a lack of proper training as a main factor; however, heavy workloads, high work intensity, stress, staff shortages, and poor work-life balance are among the other factors [9]. Emergency workplaces are notorious for being overcrowded, chaotic, unreliable, and violent [10]. This finding is also reported in our study, which has shown that 57.6% of the participants believe that a more hostile environment offered by EM is a leading obstacle in opting for EM.

Supported by the studies, EM doctors face a high risk of burnout [11]. Healthcare workers in EM have to make decisions under multiple, sometimes, severe stressors [12]. Some studies have shown that up to 35% of EM residents suffer from acute post-traumatic stress syndrome symptoms after the COVID-19 pandemic. The same study reported that early screening, referral, and treatment of high-risk physicians could be effective at addressing this risk [12]. There is evidence that some EM trainees feel that multiple barriers exist in conducting research in their emergency department. These include lack of time, skills, and cultural factors [13]. 

The use of an online survey to recruit the study participants is not free from criticism, as a considerable proportion of the targeted population could be individuals who do not have access to social media websites or would not have participated in the study. There were also other limitations that are associated with this survey method of study design. One of them is that all of the people who have been who started the survey did not complete it. First, the response rate was around 60%. Second, since this study was conducted on one single institution, more studies are needed to be carried out on other institutions as well. There is a lack of data regarding perceptions of EM in Pakistan. Hence, many of the statistics reflecting current attitudes regarding this field have been taken from African studies, where the healthcare system is set up in a similar manner to that of Pakistan.

## Conclusions

In Pakistan, many doctors and medical students are not willing to pursue their careers in EM. Nationwide educational seminars should be conducted to increase awareness and interest among doctors in this field. Moreover, more and more clinical placement opportunities should be made available for junior doctors in EM. Future research should aim to identify effective educational interventions to increase doctors’/medical students’ awareness in this field of medicine.

Pakistan lacks many tertiary care hospitals that can offer state-of-the-art training in EM. High levels of burnout, a hostile environment, lack of patient follow-up, and poor life balance are some of the factors that pose a barrier to doctors from pursuing their career in EM. Giving more stipends and increasing centers across the country that can provide training in EM are some of the steps that can not only make people opt for emergency medicine as a career but can also reduce the burden on doctors working in emergency departments across the country.

## Appendices

**Table 3:** Study questionnaire

Barriers in Opting for Emergency Medicine as a Career in Pakistan: A Cross-Sectional Survey Study									
This survey aims to understand any barriers that junior doctors and medical students may face in opting for Emergency Medicine as a career option. All your responses will be completely confidential names and personal details will not be requested.									
Section 1- Demographic Details									
1. Gender									
Male			Female				Prefer not to say		
2. Current Designation									
Medical Student	House Officer			Medical Officer		Post-graduate Resident			Others
3. Have you done a clinical placement in Emergency Medicine?									
Yes					No				
4. Do you have any close family members or friends who experienced a medical emergency?									
Yes			No				Prefer not to say		
5. Have you ever previously faced any medical emergency yourself?									
Yes			No				Prefer not to say		
6. Have you considered a career in Emergency Medicine?									
Yes			No				Maybe		
Section 2- Questions about a career in Emergency Medicine. What are the barriers you face in selecting Emergency Medicine? Please choose the most appropriate answer.									
1. I lack exposure to Emergency Medicine to make it my career choice.									
Agree			Disagree				Neutral		
2. I feel Emergency Specialists earn less than other fellows.									
Agree			Disagree				Neutral		
3. I feel doctors working in emergency medicine face a high level of burnout.									
Agree			Disagree				Neutral		
4. I feel Emergency Medicine offers a poor work-life balance.									
Agree			Disagree				Neutral		
5. I feel Emergency medicine offers a more hostile environment.									
Agree			Disagree				Neutral		
6. I feel Emergency Medicine does not offer further specialization.									
Agree			Disagree				Neutral		
7. I feel Emergency Specialists cannot work independently.									
Agree			Disagree				Neutral		
8. I feel Emergency Medicine is less satisfying due to increased mortalities and severely sick patients.									
Agree			Disagree				Neutral		
9. I feel my family will not approve of my career in Emergency Medicine.									
Agree		Disagree			Strongly disagree			Neutral	
10. I feel Emergency Medicine is less rewarding in terms of patient follow-up.									
Agree			Disagree				Neutral		
Section 3- How can we improve? In your opinion, how can the perception of Emergency Medicine be improved among junior doctors and medical students?									
